# A New Method for the Analysis of Soft Tissues with Data Acquired under Field Conditions

**DOI:** 10.1371/journal.pone.0067521

**Published:** 2013-06-24

**Authors:** Ruth S. Sonnweber, Nina Stobbe, Olmo Zavala Romero, Dennis E. Slice, Martin Fieder, Bernard Wallner

**Affiliations:** 1 Department of Cognitive Biology, University of Vienna, Vienna, Austria; 2 Department of Scientific Computing, Florida State University, Tallahassee, Florida; 3 Department of Anthropology, University of Vienna, Vienna, Austria; 4 Department of Behavioural Biology, University of Vienna, Vienna, Austria; Monash University, Australia

## Abstract

Analyzing soft-tissue structures is particularly challenging due to the lack of homologous landmarks that can be reliably identified across time and specimens. This is particularly true when data are to be collected under field conditions. Here, we present a method that combines photogrammetric techniques and geometric morphometrics methods (GMM) to quantify soft tissues for their subsequent volumetric analysis. We combine previously developed methods for landmark data acquisition and processing with a custom program for volumetric computations. Photogrammetric methods are a particularly powerful tool for field studies as they allow for image acquisition with minimal equipment requirements and for the acquisition of the spatial coordinates of points (anatomical landmarks or others) from these images. For our method, a limited number of homologous landmarks, i.e., points that can be found on any specimen independent of space and time, and further distinctive points, which may vary over time, space and subject, are identified on two-dimensional photographs and their three-dimensional coordinates estimated using photogrammetric methods. The three-dimensional configurations are oriented by the spatial principal components (PCs) of the homologous points. Crucially, this last step orients the configuration such that x and y-information (PC1 and PC2 coordinates) constitute an anatomically-defined plane with the z-values (PC3 coordinate) in the direction of interest for volume computation. The z-coordinates are then used to estimate the volume of the tissue. We validate our method using a physical, geometric model of known dimensions and physical (wax) models designed to approximate perineal swellings in female macaques. To demonstrate the usefulness and potential of our method, we use it to estimate the volumes of Barbary macaque sexual swellings recorded in the field with video images. By analyzing both the artificial data and real monkey swellings, we validate our method's accuracy and illustrate its potential for application in important areas of biological research.

## Introduction

We present in this paper a method that is applicable for the analysis of soft-tissue structures of various species living in different environmental conditions, especially when data are collected in the field. The goal is to quantify the 3D-nature of soft-tissue structures by computing their volume using landmarks found directly on the specimen of interest. This is achieved by combining existing photogrammetric and morphometric tools (PhotoModeler®5 software (www.photomodeler.com) for coordinate acquisition, and the Morpheus et al. software [1] for the orientation of the specimens) with integral mathematics for volume computations. The method uses (i) a subset of homologous landmarks for the orientation of specimens in three-dimensional physical space, and (ii) a variable number of additional landmarks, which may vary within (over time) and between subjects, for volumetric quantification. With this method, video material of soft tissues of free ranging animals can be analyzed morphometrically.

The perineal swellings of female Barbary macaques (*Macaca sylvanus*) are an example of a soft tissue structure whose 3D nature would be of interest to describe morphometrically. As with many other females of Old World monkeys, these primates develop distinct anogenital swellings during sexually active phases [2]. Swelling size changes over the female cycle and tumescence and de-tumescence were shown to be related to female sex steroids [3], [4], [5]. What information these secondary sexual characteristics convey is not yet fully understood. All published studies relating to the morphology of this feature have so far been restricted to analyses of two-dimensional data.

We chose this morphological trait as an example for the development of our method. This is because sexual swellings are soft tissues that differ in shape between individuals and species, fluctuate in volume over time, and constitute a topic of interest for many field studies. Photogrammetry is an established method by which coordinate data are derived on the basis of several two-dimensional pictures taken from different, overlapping perspectives. By marking corresponding points (“landmarks”) on two-dimensional images of a single specimen, three-dimensional coordinate data of individual points can be acquired by triangulation and can subsequently be used for three-dimensional modeling. The main advantage of such photogrammetric methods is their flexibility in use and the minimal equipment requirements for image capture (i.e., a digital camera). This makes it a suitable technology for data collection in the field. A study by Chiari et al. [6] showed the application of such a photogrammetric method using images of tortoise shells obtained in the field. As the study species is slow moving, high-quality pictures could be taken and subsequently used for landmark-coordinate acquisition. By setting landmarks at the intersections of the scutes on the carapace of the animals, geodesic distances were calculated. Results showed large deviations from measurements obtained with a flexible measuring tape, however. The authors argue that this stems from a lack of landmarks near the periphery of the tortoise shells.

A sufficient number of landmarks needs to be identified on a specimen in order to grant an adequate reconstruction as well as an accurate and precise morphological description [7]. For between- and within-individual (or species) comparisons using standard methods, landmarks must be identified reliably on all specimens across time. This is a fundamental limitation for the analysis of soft tissue structures, when applying photogrammetric techniques, as they typically exhibit a rather landmark-poor morphology. Hence, soft-tissue morphometric quantifications have focused mainly on the analysis of human facial structures (e.g. [8], [9]). The advantage of analyzing facial structures, in contrast to other soft tissues, is that landmarks can easily be assessed and reliably recovered in almost any human face. However, the analysis of other soft-tissue body parts acquired under field conditions may be of particular interest when studying, for example, living animals in natural settings. A field study of a pinniped species circumvented the lack of identifiable homologous landmarks on the bodies of the animals by using inanimate objects in the surroundings as references and successfully estimated the body mass of their study subjects [10]. This method has been further and successfully used for a number of related species [11], [12]. The applicability of this “indirect” method heavily depends on the study site and study species (e.g., slow- vs. fast-moving animals). Elephant seals often lie on the ground resting and thus maintain a specific body posture for a comparably long duration. This allows placing landmarks around the animal and taking pictures of the still subject from different angles. In addition, shores offer a multitude of inanimate landmarks to use for reference that may be difficult to find in other environments (such as savannah landscapes, deserts, etc.). Our method avoids these problems by characterizing soft-tissue morphology based on (possibly ephemeral) points intrinsic to the individual and tissue of interest and takes advantage of the flexible landmark-manipulation capabilities of geometric morphometric methods (GMM).

Landmark-based geometric morphometric methods using three-dimensional Cartesian coordinates represent a particularly important approach to shape analysis. GMM, as we know it today, had its revolutionary takeoff in the 1980’s and early 1990’s [13]. In 1982, Bookstein formulated the “foundations of morphometrics” [14] in an attempt to describe an initial framework for GMMs. Since then, methods have improved and become more sophisticated, and areas of application have expanded. GMMs allow dealing not only with 2D data, but also with variables representing shapes in three-dimensional physical space [15]. This is of particular interest for the description of many biological structures, most of which are three-dimensional. These methods allow for acquisition, processing, and analysis of shape while retaining all of the geometric information relevant to the original structures. GMMs enable assessment and visualization of shapes of specific body structures and their covariates [16], and thus statistical information about the whole shape can be obtained. Methods applying coordinate-based geometric morphometrics have been shown to be superior to alternative approaches, such as the use of derived distances or angles, in terms of their statistical properties and graphical output [16], [17], [18].

In general, 3D GMMs use sets of coordinates either obtained directly from the specimen of interest (3D digitizing technologies), or extracted from digital images of animals, specific body parts, or analogous material [7]. The production of 3D representations of subjects of interest encompasses the application of CT-scans, MRI, or laser scanning technologies.

The standard approach to GMM analysis is to identify a subset of reliably identifiable points and base all processing procedures solely on these few landmarks (“primary points”). This is largely inadequate for most landmark-poor, soft-tissue analyses since these points would not be sufficient to describe the overall shape of such a morphological structure in subsequent morphometric study. However, additional landmarks can be placed on the specimen that capture the morphology of interest and will be affected by, but not influence, preliminary alignment computations (“secondary points”). After alignment, required computations can be based on all landmarks. Morpheus et al [1] is a free software package that allows one to carry out such computations with 3D-data. Hence, it is possible to orient the entire set of points in a standardized way on the basis of the primary points by performing a General Procrustes Analysis (GPA) and available options for a final alignment to the spatial principal components of the primary points. Such standardization allows for further calculations and shape descriptions of the specimens. This method has been used in other approaches before, and has been shown to be a useful tool for morphometric analyses (e.g. [19]). Note that in our application, the GPA alignment is a “dummy” operation – there is no need to align an object with itself. It is performed to take advantage of its ability (as implemented in Morpheus et al.) to fix the final orientation based on the major axes of variation in the mean landmark configuration, which in the case of a single individual is simply the set of primary points of that individual. To our knowledge this has never been applied to the analysis of soft tissues on fieldwork-based data.

For the development and validation of our method we manufactured artificial swellings mimicking sexual swellings of female Barbary macaques at three different stages: fully engorged, medium tumescent, and not swollen. In addition, we constructed a geometric reference object (a stepped pyramid) of known dimensions with which to further gauge the accuracy of our measurements. We describe the development and validation of the method in the following steps: (1) we show landmarking and orientation procedures of the artificial swellings using the Photomodeler®5 and Morpheus et al. software tools, (2) we compute the volumes of the three artificial swellings using custom (MATLAB) code, and (3) we validate our method by demonstrating its accuracy with respect to distance and volumetric measurements using the geometric reference object and the artificial swelling models and examine the effects of replication and landmark number on volume estimates. The first validation step is to show that measurements of distance are obtained accurately independent of the camera angle/tilt. Second, we demonstrate that when replicating the setting of landmarks and orientation of the artificial swellings, volume results for individual artificial swellings are comparable. Furthermore, we bootstrap our landmark coordinate data to illustrate the impact of the number and density of landmarks on volume estimates. Lastly, we show that the volumes obtained through our new geometric morphometric method correspond to those gained by a well-established surface scanning method. To illustrate a possible application of our method, we compute the volumes of real Barbary macaque perineal swellings using video material obtained from fieldwork.

## Methods

### 1. Morphometric analysis of artificial swellings

**Figure 1:**
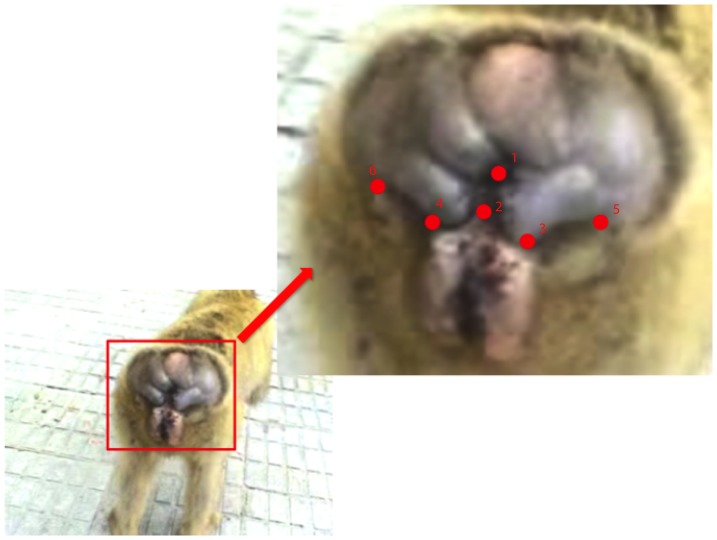
Identifying homologous points. The six homologous landmarks on the perineal swelling of a female Barbary macaque. In addition to the primary landmarks used for transformation parameter estimation, a number of secondary landmarks were also digitized for use in volume estimation. These might include scar or wrinkle termini or even dirt or trash affixed to the surface of the swelling. The only requirement of these landmarks is that they are visible and positionally stable in multiple views of the individual at the time the images are recorded.

In geometric morphometric analyses, a set of points called landmarks describes specimens. Usually landmarks are set on all specimens on corresponding, easily identifiable, anatomically defined locations, in order to translate, rotate and scale them to a common coordinate system as required for any further shape analysis. As mentioned before, one core issue with the analysis of soft tissues is the lack of easily identifiable landmarks that can be found on all specimens at any given time in the same location. Therefore, we use a limited number of points (“primary landmarks”) that can be identified on all specimens at any time for orientation, and additional (possibly unique or ephemeral) landmarks (“secondary points”) for volume computations. For the analysis of anogenital swellings of Old World primates we identified six homologous points (Figure 1): (1) the middle of the anus, (2) the center of the upper end of the vaginal entrance, (3+4) the right and the left point where the medial side of the callosities, the labia and the perineal swelling meet and (5+6) the two points where the distal part of the callosities and the perineal swelling meet (left and right). These six points qualified to define a plane for orientation of the model because they can be found on any female and they do not change their relative location or orientation over time in different individuals. Furthermore, and of particular importance for this study, the location of these primary landmarks and their relation to each other are independent of the swelling state. Neither the callosities, nor the anus or the upper point of the labia are subject to spatial fluctuations.

#### 1.1. Building artificial swellings

**Figure 2:**
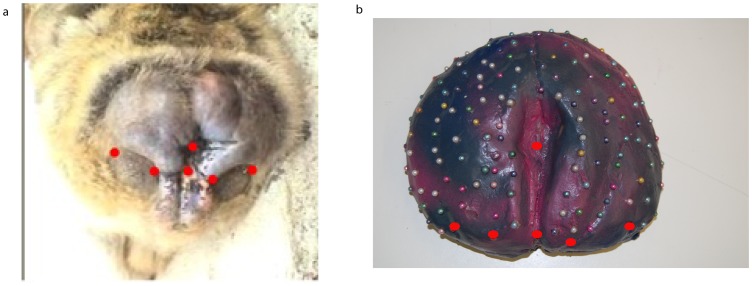
Homologous points on a real macaque swelling and on an artificial swelling. The artificial swellings were manufactured in a way that they mimic the nature of real ones. The six homologous points are indicated by red dots.

Three artificial swellings mimicking perineal sexual swellings at different stages were produced: one representing a maximally tumescent swelling, one moderately swollen, and one in a non-engorged state. These artificial swellings were manufactured out of clay. Anatomical features, such as the anus or bulges above the callosities, were represented in a swelling-state typical manner. Thin, metal pads were placed tightly next to each other leaving no gaps in the clay model, which was then covered with plaster to create a negative mold. The pads were necessary to be able to separate the clay model from the plaster once it had dried. A wire held the two halves of the plaster negatives together. Melted wax was applied in layers in the plaster mold. When the wax layer was about five centimeters thick, plastic material was used as a filling element. The artificial swellings were “closed” by another wax layer. In this way artificial swellings with wax coverage were produced. They were then colored with blue and pink paint to mimic coloration of a typical Barbary macaque swelling. Colored pins (90–134) were used to indicate landmarks (on a real monkey, scars, scratches, small bits of debris, etc. would be used). All pins were inserted into the artificial swelling in a way that the heads contacted the surface of the artificial swelling. Red pins were used to define homologous landmarks that could be found on any real Barbary macaque sexual swelling, namely: the middle of the anus, the center of the upper end of the labia, and the beginning and end of the callosities on either side. The rest of the pins mimicking other landmarks were inserted in a haphazard manner, representing locations on the whole artificial swelling (Figure 2).

#### 1.2. Landmarking and orientation

The artificial swellings with pins mimicking landmarks were photographed (Nikon D40, 10 Megapixel resolution) from different angles. The camera was calibrated in the program PhotoModeler® 5. Three single images (a frontal and two laterals with respect to the model surface) were used and processed in PhotoModeler®5. On the frontal image of the artificial swellings, landmarks were set by using the marking tool, where each pin on the frontal image represented one landmark (90 – 134 landmarks depending on the artificial swelling type: large, middle, small). Subsequently, the referencing tool was used to associate the corresponding points on the other two images and the x, y, and z coordinates for each landmark were calculated. Additionally, landmark-referencing accuracy was evaluated. The program gives total error (log scale) values that indicate the "goodness of fit" of diverse input data (camera parameters, mark locations and 3D points). Values below 0.5, as suggested by the program manual, were accepted as sufficiently precise. Landmark data of x, y and z coordinates were exported in RAW format. A text file containing the 3D coordinates from the RAW file was created in the NTSYS-pc format [20] suitable for import into Morpheus et al.

**Figure 3:**
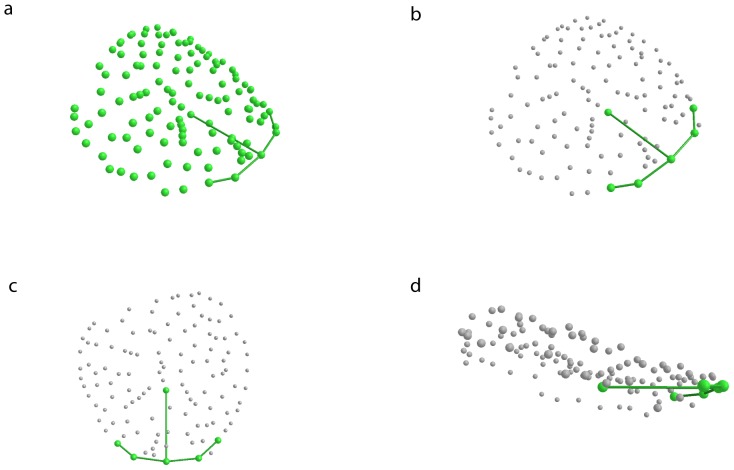
Orientation of the artificial swelling reconstructions. Coordinate data read into Morpheus et al. and plotted in 3D space. (a) All landmarks from photogrammetric reconstruction. (b) All but the first six anatomical landmarks demoted to secondary points. (c) Representation of alignment of the six anatomical landmarks to their spatial principal components (view of xy, i.e., PC1PC2, plane from above). (d) Representation of lateral view after alignment. Secondary points (gray) were used in the volume estimation based on their position (xy) and height (z) above the plane. Not all figures to the same scale. Symbol sizes, especially in (d) somewhat distorted due to plot perspective and magnification.

In Morpheus et al. [11], further processing of 3D coordinate data included the definition of primary and secondary landmarks via batch files. Primary points were the six homologous points described earlier. All remaining landmarks were treated as secondary points (for a visualization of the following process see Figure 3). After demoting the secondary landmarks, a General Procrustes Analysis (GPA) with a final orientation defined to be the spatial principal component axes of the primary points resulted in an orientation of the reconstruction along the plane through the six homologous points. Hence, the x and y axes correspond, in general, to the best-fitting (in the least-squares sense) plane perpendicular to the direction of swelling. In the following step, actual size information was restored in order to include it in the computation of volumes. After the individual artificial swelling models were superimposed and oriented, as well as the size of the reconstructions restored (see below), the secondary points were promoted to primary points. The coordinates of the new, oriented artificial swelling reconstruction were then used for further morphometric and volumetric analyses.

#### 1.3. Volume computation

To obtain actual volume data of the artificial swellings from the coordinate data of the reconstructed models, it was necessary to scale the data with a distance of known length. For this purpose, we measured the distance between the third and the fourth homologous points on the artificial swellings and on the reconstructed point configuration. By rescaling by the ratio of these measurements, we obtained coordinate data corrected to provide true distance and volume measurements. As a first step, the set of coordinate data of the artificial swelling models were positioned similarly in physical space (as described above). Then, the bottom plane for each artificial model was computed as the plane parallel to the x-axis that passes through the lowest z value of the set of all landmarks. A polygon was then calculated in relation to the horizontal plane and its volume was computed as follows.

The height of each reconstructed model of the artificial swellings was interpolated into a 60×60 grid, using cubic interpolation of all of the reconstructed landmark coordinates, to obtain a smoothed surface of the reconstruction. From this surface, a rectangular prism for each cell of the grid was obtained by taking average heights across these cells of the artificial swelling model as the height of the prism with respect to the previously computed plane. Volumes were computed for individual rectangular prisms and then added together to obtain the overall volume of the artificial swelling. This method was implemented in MATLAB (www.mathworks.de/products/matlab/) and was used to compute the volumes of three artificial swelling reconstructions.

### 2. Validation of the method

For validation of our method, we performed the following computations (Table 1), we (1) calculated the accuracy of our measurements in space using a step pyramid of known dimensions and compared the real measures to those obtained through the reconstruction of the pyramid; (2) performed replicate analysis of our artificial swellings; (3) made a bootstrap analysis of the model-volume estimates with respect to landmark density and observer; (4) computed volumes from high-density surface-scans of the artificial swellings and compared the volume results gained from this validated method to ours.

**Table 1:** Overview to the validation procedure of the method.

Validation step	Method	Validated quantities
Precision of distance measurements	reconstruction of a wooden step pyramid with analysis of length and height measurements	distance measurements in the three-dimensional physical space, irrespective of angles and tilts of the camera used for picture taking
Replication of volume computations for the artificial swellings	repeating the process of picture taking, landmarking for coordinate data acquisition, orientation of the reconstructions in the 3D space, and volume computations for the three artificial swellings	the repeatability of volume measures obtained by the application of the GMM
Bootstrapping of volume results	sampling different percentages of landmark coordinate data from 15 landmark data sets (original reconstructions and their replications) and calculating the corresponding volumes	effects of landmark number and density on volume results
Surface scanning of the artificial swellings	scanning the surfaces of the three artificial swellings and computing their volumes in AMIRA	validating the accuracy of the volume results obtained through the photogrammetric approach by comparing it to volumes derived from a well-established method

The validation of the method was divided into several steps. Here we give a description of the individual steps explaining the reasoning behind them.

#### 2.1. Precision of distance measurements

**Figure 4:**
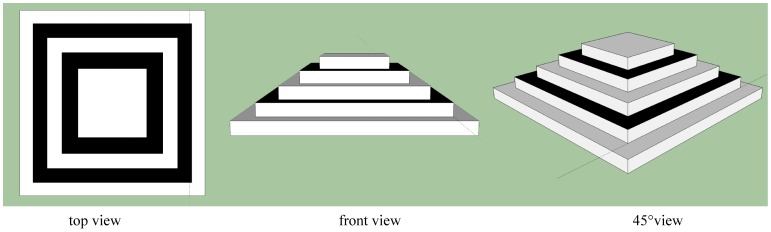
Perspectives on the wooden pyramid. Pictures of the wooden pyramid were taken from three different perspectives: from above, in front and from 45 degrees.

We did not apply a standardized protocol for picture taking of the object of interest. Hence we aimed at evaluating the precision of distance measurements obtained, independent of the perspective used to take a photograph of the object of interest. For this purpose we constructed a wooden step pyramid consisting of five levels. Each level was build of a square wooden block of 1.8 cm height. The lowest plane was 30×30 cm in length and width, the second 25×25 cm, the third 20×20 cm, the fourth 15×15 cm and the fifth 10×10 cm. Images of this pyramid were then taken (Nikon D40, 10 Megapixel resolution), once from above (standing above the pyramid taking pictures from left, right and center), once from in front of the pyramid (camera on same level as basis of the pyramid), and once from 45 degrees (Figure 4). Three individual pictures of different angles were selected and pyramid corners landmarked in Photomodeler®5. Coordinate data were exported in RAW format and then transformed into an NTSYS-pc-file. Code written in MATLAB merged the coordinate data obtained from the different perspectives and then calculated all lengths, widths and heights of all planes (lower and upper plane of all levels).

#### 2.2. Replications of volume calculations

The three artificial swelling types (large, middle, and small) were analyzed repeatedly, each five times, resulting in a total of 15 reconstructed models. Each replication consisted of a new landmark setting procedure in Photomodeler®5, orientation of the models and scale restoration in Morpheus et al., rescaling of the coordinate data and calculation of the volumes with our MATLAB code. This was to demonstrate that volume results for the artificial swellings were repeatable.

#### 2.3. Bootstrapping and analysis of variances

The total number of landmarks, as well as landmark density, differed across the artificial swelling types. To assess the influence of numbers and density of landmarks on estimate variablity, we estimated volumes with many replicates using random subsets of landmarks. Subsets consisting of 25, 50, 75 and 90 percent of the total number of landmarks were used. We hypothesized that an increasing number of landmarks would lead to a decrease in overall variance within artificial swelling models.

All data derived from the analyses of the original models of the artificial swellings, and their replications (3 artificial swellings each landmarked five times) were used in this validation step. Using the coordinate data of a model in a PYTHON (www.python.org) script, the first six (homologous) landmarks were retained and another 25, 50, 75 or 90 percent of coordinate data were randomly selected without replacement and retained for analysis. This process was repeated 100 times for each of the 15 original reconstructions and each resulting coordinate set was analyzed. Variances and mean volumes of each of these new sets of coordinates were again calculated with the MATLAB code for volume computations. To estimate the effects of artificial swelling types and the number of landmarks on the results, we used a Generalized Estimating Equations (GEE) model (see description of statistics below). All significance values were calculated using sequential Bonferroni corrections.

#### 2.4. Surface scans

To assure that our volume results represent adequate relations between artificial swelling types, we scanned and measured their surfaces. 3D images of the three artificial swellings were produced using a triTOS surface scanner (Breuckmann GmbH). The scanner base length was 300 mm and the measuring range was 675 mm. The orientation of the artificial swelling models was standardized for scanning.

From the raw scans, STL files were obtained that could be loaded into AMIRA 5.4.2. (www.amira.com). We cut a surface through the lowest point of the artificial swelling model and created a base surface. Subsequently, the volume above this surface was calculated (units used were voxels). Relations between the large and the middle, the large and the small, as well as the middle and the small artificial swelling were calculated and compared to the relations obtained with the average volume results of each artificial swelling type from our method.

#### 2.5. Statistics – Generalized Estimating Equation (GEE) Model

All statistics were computed using SPSS 19. We used Generalized Estimating Equation (GEE) models with a normal probability distribution and an identity link function. All testing of artificial swelling effects was performed with type III sum of squares. Post-hoc comparisons of significance values were sequential Bonferroni corrected to account for repeated use of the data. For calculations on the replications of the artificial swelling reconstructions, we had the volume as our dependent variable, the model type as the subject effect, and the repetition as a within-subject effect. For the bootstrapped data we used the artificial swelling type as a subject effect. The percentage of landmarks added and replicates were included as within-subject effects. For testing effects on mean volumes and variances in volumes, we included the individual artificial swellings as subject effects and the percentage of landmarks as within-subject effects.

### 3. Analysis of real monkey swellings

After testing and validating the method using artificial swellings, we applied it to swellings of actual female Barbary macaques. The conduct of the study was approved by Dr. John Cortez and Dr Eric Shaw from the Gibraltar Ornithological and Natural History Society (GOHNS). No invasive methodologies were used in this study, only behavioral observations and videography of the monkeys and collection of urine samples. Videos (filmed with a Sony Mini DV Digital Handycam) of female swellings were recorded in two different groups of macaques inhabiting the Rock of Gibraltar. For focal individuals, videos were taken every second day alternating between the groups. Data collection spanned over the reproductive period from October 2005 to March 2006.

To test if our method is applicable to real biological structures with data collected in the field, we selected three individual females from the video archive for morphometric swelling analysis. For each of the three females, images of a fully tumescent and a flat swelling state were selected. To improve landmark reliability, a series of five images (instead of only three) per individual and swelling state were retrieved from the video sequences. To gain 3D coordinate data, two people set the landmarks on each of the swelling pictures independently using the PhotoModeler®5 software. Landmarking, orientation, and rescaling were performed as described earlier for the artificial swellings. Since we did not have a scaling factor for the coordinate data we assumed a standardized distance of five centimeters between the third and the fourth landmark for all females.

## Results

### 1. Morphometric analysis of artificial swellings

**Figure 5:**
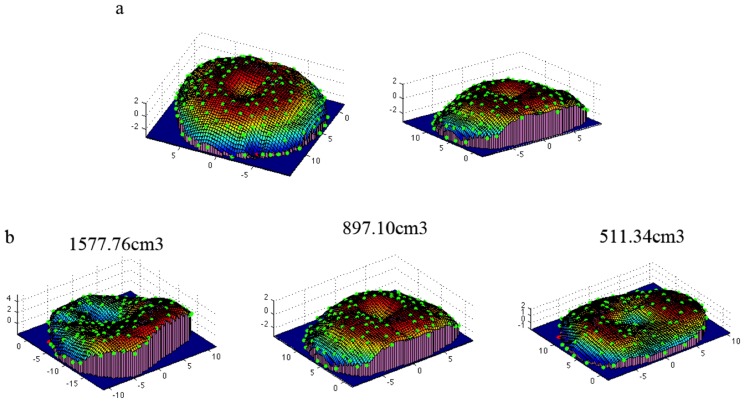
Reconstructions of the models. a) 3D reconstruction of the middle size artificial swelling from two perspectives. Green dots represent the original landmarks and red dot is the landmark with the lowest z-value used to compute the bottom plane (in blue). The rectangular prisms are shown in purple and the smoothed surface of the model as a colored mesh. b) 3D reconstructions of three artificial swelling models (large, middle and small) and respective volume results.

Following the method described earlier, we computed the volumes of the three artificial swellings. Figure 5a shows an example of the 3D reconstruction of the middle artificial swelling obtained with the described process. Figure 5b shows the 3D reconstruction of all three artificial swellings with their respective volume estimates. The red dots indicate the respective lowest points of the models, which were used to create the bottom plane for volume computation.

**Figure 6:**
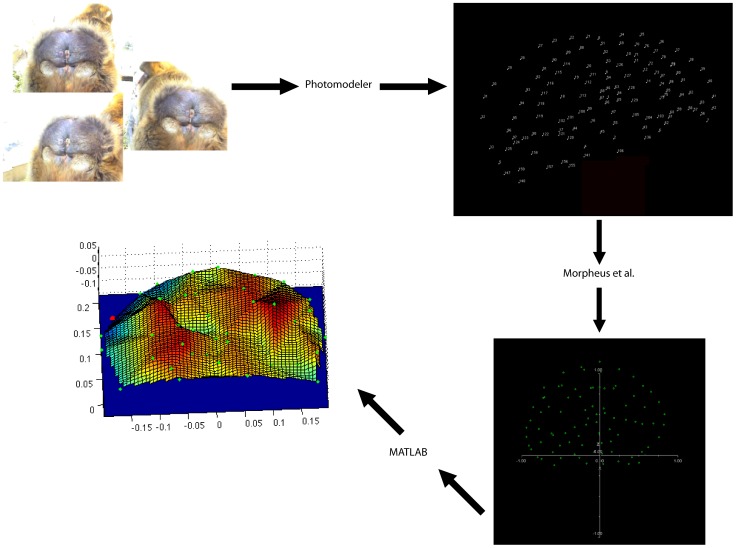
Workflow. This figure illustrates the workflow from image selection, through coordinate reconstructions, landmark-configuration alignment, and, finally, volume estimation.

In the following chart (Figure 6), we show a workflow of the individual steps that should be followed when applying this method to any soft tissue structures (see also Method section 1.2 and 1.3). The code for volume computations is available from RSS.

### 2. Validation of the method

#### 2.1. Precision of distance measurements

Testing for accuracy of distance measurements using the wooden pyramid (for details see the Methods) we obtained a root-mean-square error of 0.54% for lengths and widths, and 5.47% for the (much smaller) heights. An overview of all distance measurements obtained from the different perspectives is given in Table 2.

**Table 2:** Distance measurements of the reconstructed pyramid.

plane	1	2	3	4	5
side length 1	30	24.9196	19.9942	14.9347	9.9614
side length 2	30.1241	25.2138	20.1101	14.947	9.9842
side length 3	30.2243	25.2952	20.0746	14.9831	10.0634
side length 4	30.1959	25.0874	20.1495	15.0214	9.8928
side length 5	29.8457	24.9114	19.9776	14.9661	9.8988
side length 6	30.3172	25.2103	20.4224	14.726	9.6928
side length 7	30.6273	25.4868	20.5491	15.2583	10.0916
side length 8	30.1939	25.2378	20.2423	15.1245	10.0861
**avgErrorPercentage**	**0.6368**	**0.6811**	**0.9499**	**0.0325**	**0.4112**
height1	1.6977	1.7095	1.639	1.6363	1.609
height 2	1.6927	1.6497	1.6017	1.6703	1.5417
height 3	1.7171	1.6709	1.7757	1.8193	1.8201
height 4	1.6868	1.7242	1.6951	1.795	1.8807
**avgErrorPercentage**	**5.6353**	**6.1903**	**6.7848**	**3.8761**	**4.8399**

In the columns, length (side length1 to 8) and height (height1–4) measurements of the individual planes of the pyramid (1–5) are given. Actual distances were 30, 25, 20, 15, and 10 cm for lengths, and 1.8 cm for heights. Average error percentages of measurements are given and range from 0.0324 to 0.9499% for length measurements and from 6.7848 to 3.8761% for height values.

#### 2.2. Replication of volume computations for the artificial swellings

The results of the replicated volumes (for details see Methods) are shown in Table 3.

**Table 3:** Volume results of artificial models from 5 analyses.

	large (cm3)	middle (cm3)	small (cm3)
**replication 1**	1577.76	897.09	511.34
**replication 2**	2036.63	905.1	531.74
**replication 3**	2307.6	781	455.44
**replication 4**	1442.84	806.64	563.39
**replication 5**	1689.92	821.43	599.46
**mean**	1810.95	842.252	532.274
**SD**	354.3033637	55.70155985	54.36944252

The table presents the volume results for the large, middle and small model obtained from five analyses procedures. Each replication involved picture taking, landmarking, orientation of the model and volume computation.

A GEE model revealed significant differences over all artificial swelling types (Wald χ^2^ = 161.650, df = 2, p<0.001). Pairwise comparisons showed volume differences between the large and the middle, the large and the small, as well as between the middle and the small type. Between replications no significant effects were found (Wald χ^2^ = 3.131, df = 4, p = 0.54).

#### 2.3. Bootstrapping and analysis of variance

**Figure 7:**
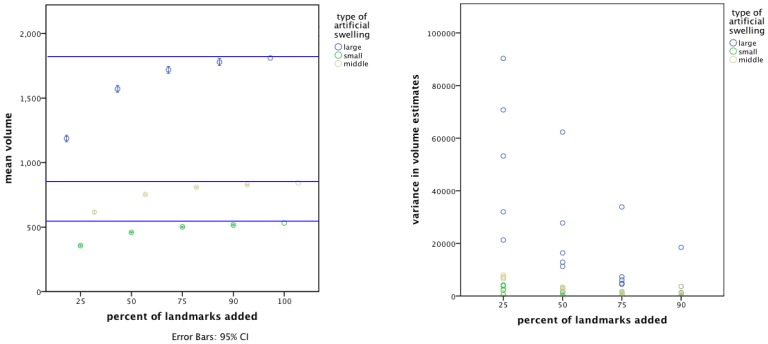
Means and variances of the bootstrapped volumes per model type and landmarks added. a) The mean volume was affected by the type of model (large, middle, or small) as well as by the percentage of landmarks added. With an increasing number of landmarks the mean volume asymptotically increased. The blue lines indicate the values of the mean volumes of the five replications with 100% of the landmarks for each artificial swelling size. b) Variances in volume results were influenced by the type of model and the number of landmarks. As the percentage of landmarks added increased, the variance in volume results decreased.

Bootstrapping analysis showed that both the type of the artificial swelling (Wald-χ^2^ = 174.211, df = 2, p<0.001) and the percentage of landmarks added (Wald-χ^2^ = 261.364, df = 3, p<0.001) affected the resulting volume values (Figure 7a). Furthermore, the interaction between the type of swelling and the percentage of landmarks added was shown to be significant (Wald-χ^2^ = 62.035, df = 6, p<0.001). This means that the effect of the number of landmarks used depended upon the size of the specimen. Pair-wise comparisons revealed significant differences between volumes of individual model types and between landmark percentages, respectively. An increasing number of landmarks included in the calculation led to an increase in the resulting volumes of the models.

Variances in volume data (Figure 7b) were significantly affected by both the type of artificial swelling (Wald-χ^2^ = 19.355, df = 2, p<0.001) and percentage of landmarks, (Wald-χ^2^ = 45.830, df = 3, p<0.001) as well as by their interaction (Wald-χ^2^ = 144.576, df = 6, p<0.001). The more landmarks that were added for artificial swelling reconstructions, the more the variance between volumes decreased. The larger the swelling, the higher was the variance in volumes. Post hoc tests revealed that this was true for comparisons between all artificial swelling types (large to middle: mean difference = 21362.65, df = 1, p = 0.003; large to small: mean diff = 22659.49, df = 1, p = 0.002; middle to small: mean diff = 1296.833, df = 1, p = 0.003), as well as between all percentages of landmarks added (see Table 4).

**Table 4:** Post hoc pair-wise comparisons of the GEE for volume variances and percentage of landmarks.

		percentage of landmarks added			
		25	50	75	90
**percentage of landmarks added**	25		(6321.86, 15253.50)	(9547.87, 23414.29)	(10664.42, 26665.80)
	50	10787.68 (1787.97)		(2661.64, 8725.18)	(3737.55, 12017.31)
	75	16481.08 (2691.64)	5693.41 (1266.41)		(791.59, 3576.46)
	90	18665.11 (3032.57)	7877.43 (1847.00)	2184.02 (710.44)	

The lower diagonal shows the absolute values of the mean differences over the standard deviation (in parentheses) and the upper diagonal shows the lower and upper confidence limits (95% CI). All significance values were sequential Bonferroni corrected and were p<0.002.

#### 2.4. Surface scans of the artificial swellings

Applying both the surface scanning and the photogrammetric approach, we found that the middle artificial swelling was about half the size of the large one (surface scans: 46.57% and GMM: 46.51%) and the small one slightly more than half the size of the middle one (surface scans: 62.68% and GMM: 63.20%). The small type was only about 29% of the large swelling (surface scans: 29.19% and GMM: 29.39%). Crucially, these volumetric relations were found independent of which method was applied for volume calculations.

### 3. Analysis of real monkey swellings

**Figure 8:**
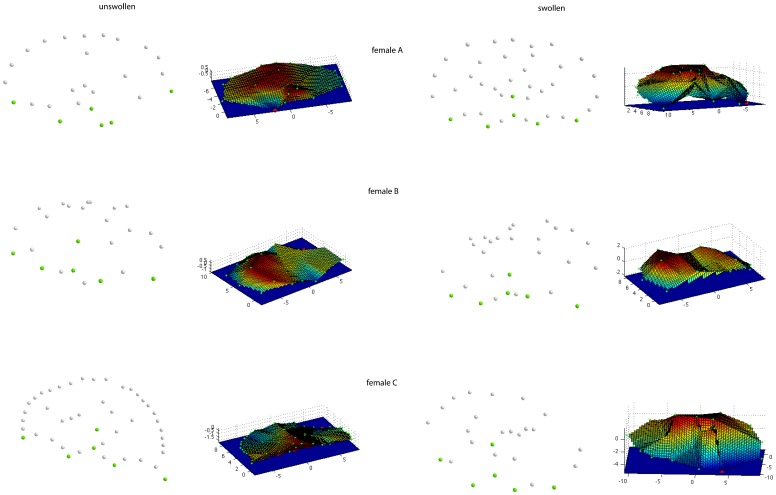
Point-clouds of oriented landmark data and 3D-reconstructions of Barbary macaque anogenital swellings. On the left side, landmark data (green dots represent the homologous points, grey dots show secondary points) and 3D-reconstructions for female A, B, and C in an unswollen state are depicted. The right side shows landmark data and 3D-reconstructions of swollen swelling states.

The volumes calculated on the basis of the landmarking from two people, and the respective numbers of landmarks used are shown in Table 5. Although the number of landmarks set was comparable between landmarkers, different people may vary in their landmarking style, leading to different absolute volumes. The relative changes from an un-swollen to a swollen state are comparable across the results derived from two people landmarking the images. Figure 8 shows the landmark data after superimposition, and the 3D-reconstructions of the swellings for each female in two swelling states (unswollen and swollen).

**Table 5:** Comparison of volume results of Barbary macaque anogenital swelling volumes obtained by the landmark setting of two people.

female	swelling state	landmark set 1 volume (cm^3^)	landmark set 2 volume (cm^3^)
A	swollen	724.73 (33)	604.69 (48)
A	unswollen	118.31 (18)	41.79 (20)
B	swollen	313.85 (27)	227.62 (23)
B	unswollen	174.67 (21)	81.29 (19)
C	swollen	738.19 (40)	773.77 (43)
C	unswollen	139.24 (31)	142.67 (21)
**increase in volume (cm^3^)**			
A	swollen - unswollen	606.42 (15)	562.90 (28)
B	swollen - unswollen	139.18 (6)	146.34 (4)
C	swollen - unswollen	598.96 (9)	631.10 (22)

The table shows volumes and number of secondary landmarks set (in parentheses), and the change in volume with the difference in number of landmarks set between swelling states (in parentheses). Although absolute volume results do not coincide, the increase in volume from the unswollen to the swollen state was found to be comparable across landmark setters.

## Discussion

Our newly developed method enables the volumetric analysis of soft tissues on the basis of photogrammetric data acquisition. Hence, 3D information about the morphology of soft tissues is calculated with coordinate data derived from images collected in the field with minimal equipment requirements. Using artificial swellings, we demonstrated the application and validation of our approach. One core element of shape description and shape analysis is the use of landmark data. All information not relevant to the shape of a specimen is mathematically removed and only shape variables (invariant to location, orientation, and scale) are used for statistical and comparative analysis as well as for graphical representation [14], [15], [16]. To achieve this, superimposition methods are used. In our case, we used numerous points to describe the shape of the structure, but only six homologous points on our objects of interest for achieving a final orientation of the whole specimen to the PC axes of those homologous points. We restored one non-shape variable (size) in the data since size is valuable information in the calculation of absolute volumes. Rotational and translational information were removed from the data by General Procrustes Analysis procedures. Using custom written code we showed how the volume of soft tissue structures can be calculated on the basis of only a few pictures that can easily be obtained in the field.

Both the visual representation of the reconstructed artificial swellings, as well as the results of the relative volumes of the small, middle, and large type were as expected: the visualization of the reconstructions mirrored the actual appearance of the artificial swellings well. The volume obtained for the large swelling was notably larger than the one for the middle and the small types, respectively. Thus, the method delivered results as would be expected. Therefore we proceeded to perform a validation of the method.

First, we evaluated the accuracy of distance measurements obtained from taking pictures of an object from different perspectives. For length measurements, the overall root mean square error rate was below one percent, and for (much smaller) heights below six percent. Considering the extreme differences in perspective (from above, in front, and at 45 degrees of the pyramid) these results show that the measurements we obtained were relatively accurate and precise. Taking pictures from extreme angles should be avoided, however, when looking for subtle changes across time in a morphological trait.

In a further validation procedure, we replicated the whole process of analysis. Again, relative volumes for individual artificial swelling types followed the expected pattern: volumes of the large artificial swelling were constantly larger than those obtained for the middle type, and small-swelling volumes less than those of the middle type. A statistical analysis showed that differences between artificial swelling types were significant, but differences between replications of individual swellings were not. However, replications revealed differing absolute volumes for individual models. This was particularly true for the large artificial swelling. One potential explanation for this phenomenon could be that the pictures of the swellings were taken from extreme perspectives. Hence, the lowest points used for the definition of the cutting plane for volume computations differed across replications. To be able to monitor subtle changes in a morphological soft-tissue trait, the results the method delivers may need to be more fine grained. This could be achieved by keeping the lowest point constant over time, and hence reduce variation in volumetric results between replications. Another potential explanation for the differences in absolute volume results may be that different “landmarking styles” were applied between replications. Different numbers and varying density of landmarks could impact the absolute volumes obtained.

To test this, we bootstrapped the coordinate data for all artificial swellings and their replicates. The larger the artificial swelling, the more landmarks were placed on its surface, and the more points used to approximate the surface, the larger the volumes. This is not surprising as on a larger surface more points can be potentially identified. An analysis of variance of the bootstrapped volume results revealed that the variance in volumes decreased with the number of landmarks sampled. Therefore, we suggest performing a “pilot-landmarking study” to evaluate the density and approximate number of landmarks required to adequately describe a specimen of interest. By analyzing the variances in volume results across replications an optimal “landmarking-style” can be inferred.

By comparing the volume results obtained through our photogrammetric approach with those found by surface scanning of the artificial swellings, we showed that the new method reliably reconstructs the 3D nature of our specimens. The relative volumes of the three different artificial swelling types were consistently found applying either method. This indicates that our method allows for accurate volumetric analysis and yields the same results as another well-established method that is not suitable for the field.

After validating the method using artificial swellings under controlled laboratory conditions, we applied it to video stills of Barbary macaque swellings that were collected in the field. Although we aimed at mimicking natural swellings as closely as possible when manufacturing the artificial swellings, we acknowledge that natural swellings are structurally more complex. Furthermore, videos in the field were obtained using a low-resolution video camera, while artificial swellings were photographed using a high-resolution camera. This may result in a larger error rate for the real monkey-swelling volume results. However, angles from which the pictures were taken and distances to specimens of interest were comparable for the artificial and the natural setting. The results for the real swellings obtained by different individual’s landmarking differed from each other although landmark numbers were similar. The relation between swelling states across observers were comparable. This hints again to the importance of having a single person landmarking the specimens, or adopting a thorough landmarking protocol, as not only the number of landmarks used to describe a specimen may influence the volume results, but also different landmarking styles.

Potential users of this method should consider several points in order to improve and optimize the results. When it comes to taking photographs, one major advantage of the presented method is that only a very limited number of images is needed for reconstructing a biological specimen. Although such reconstructions are possible with as few as three single pictures, we have found that including five instead of three film still images improved the precision of the data gained from this analysis step (as indicated by the error measures given by Photomodeler®5). Furthermore, angles from which single pictures are taken should not differ too much, and should overlap to a great extent. It is already known from other morphometric methods (http://insight3d.sourceforge.net/) that the quality of the reconstruction increases with a greater overlap of angles between individual pictures. Thus, including more film stills (that are easy to collect using a video camera instead of photo cameras) with relative high overlap in the reconstruction process produces better results.

The software Morpheus et al. [1] allows for demoting and promoting specific points used to describe a specimen, and subsequently perform a GPA (to orient the specimens) on the basis of a limited amount of coordinate data (in our case just six landmark points). This is crucial since soft tissues often do not exhibit a great number of conspicuous homologous points. All further landmarks representing a specimen at a given location and point in time can be arbitrarily distributed. However, the total number of landmarks may play a role in the results. Therefore, we emphasize the importance to pre-test how many points most reliably describe a specimen and how densely landmarks should be distributed before conducting further analyses. The density of landmarks should be commensurate across specimens for later comparison between individuals. When different people landmark different specimens, it is advisable to unify the landmark-setting procedure (the way landmarks are placed on a specimen, e.g. density, perimeter of the specimen) in order to gain comparable shape variables.

A further essential issue for computation is the definition of the lowest plane above which the volume of an object is calculated. For the demonstration with the artificial swellings, as well as for the application to real macaque swellings, the cutting surface was placed through the lowest point on the specimen. Depending on the object of interest different cutting planes may be reasonable. Again, pilot testing to identify an appropriate cutting plane should be performed prior to the actual study.

A possible application of the method presented here may be to clarify the functional aspects of anogenital swelling expressions in the context of reproductive behavior in different primate species since, for the first time, it allows the analysis of this three-dimensional trait in three-dimensional physical space. As stated by Zinner et al. [21], there is still no conclusive empirical explanation available for this very conspicuous feature expressed by many primate species. This could, at least partly, be due to the fact that all studies so far rated or described these soft tissues by length and width measures only. Our new 3D methodological approach might help to answer at least some of these questions in the future.

More generally, these results may induce activities in diverse research fields where functional aspects of soft tissues changing their expression rates over time are studied. For example, changes in body volume across seasons or in the course of a lifetime or monitoring of body structure changes in free ranging animals for nutrition and welfare studies could be quantified using the method detailed here.

As Adams, Rohlf, and Slice [15], stated in their review on GMMs, the use of 3D data in modern morphometrics is still deficient. This is particularly problematic since many biological shapes are three-dimensional and, thus, should be described in this space. Our method contributes to the enhancement of GMM for future use in the natural sciences and should help pinpoint the role and importance of shape and volumetric information in three-dimensional space.

## References

[1] Slice DE (1998) Morpheus et al.: Software for morphometric research.Revision 01-31-00, Department of Ecology and Evolution, SUNY, Stony Brook, NY.

[2] Dixon AF (1983) Primate Sexuality: Comparative Studies of the Prosimians, Monkeys, Apes, and Human Beings. Oxford University Press.

[3] Wallner B, Dittami J, Wallis J (2006) Influence of perineal swellings on behavior and stress reaction in levonogestrel implanted *Macaca sylvanus* females. Neuroendocrinology Lett 27(1–2): 253–256. 16648796

[4] Möhle U, Heistermann M, Dittami J, Reinberg V, Wallner B, et al (2005) Patterns of anogenital swelling size and their endocrine correlates during ovulatory cycles and early pregnancy in free-ranging Barbary macaques (*Macaca sylvanus*) of Gibraltar. Am J Primatol 66: 351–368. 16104035 10.1002/ajp.20161

[5] Küster J, Paul A (1984) Female reproductive characteristics in semifree-ranging Barbary macaques (*Macaca sylvanus* L. 1758). Fol Primatol 43: 69–83. 10.1159/000156173 6519600

[6] Chiari Y, Wang B, Rushmeier H, Caccone A (2008) Using digital images to reconstruct three-dimensional biological froms: a new tool for morphological studies. Biol J Linnean Society 95: 425–436.

[7] Zelditch ML, Swiderski DL, & Sheets HAD. Practical Companion to Geometric Morphometrics for Biologists: Running analyses in freely-available software. http://booksite.elsevier.com/9780123869036/content/Workbook.pdf

[8] Hennessy RJ, Moss JP (2001) Facial growth: separating shape from size. European J Orthodontics 23: 275–285. 10.1093/ejo/23.3.275 11471270

[9] Hennessy RJ, Kinsella A, Waddington JL (2002) 3D Laser Surface Scanning and Geometric Morphometric Analysis of Craniofacial Shape as an Index of Cerebro-Craniofacial Morphogenesis: Initial Application to Sexual Dimorphism. Biol Psychiatry 51: 507–514. 11922887 10.1016/s0006-3223(01)01327-0

[10] de Bruyn PJN, Bester MN, Carlini AR, Oosthuizen WC (2009) How to weigh an elephant seal with one finger: a simple three-dimensional photogrammetric application. Aquat Biol 5: 31–39.

[11] Postma M, Bester MN, de Bruyn PJN (2013) Age-related reproductive variation in a wild marine mammal population. Polar Biol. DOI 10.1007/s00300-013-1298-4

[12] Waite JN, Mellish JAE (2009) Inter- and intra-researcher variation in measurement of morphometrics in Steller sea lions (*Eumetopias jubatus*). Polar Biol 32: : 1221–1225. DOI 10.1007/s00300-009-0649-7

[13] Rohlf FJ, Marcus LF (1993) A revolution in morphometrics. Trends Ecol Evol 8: 129–132. 21236128 10.1016/0169-5347(93)90024-J

[14] Bookstein FL (1982) Foundations of Morphometrics. Ann Rev Ecol Syst 13: 451–470.

[15] Adams DC, Rohlf FJ, Slice DE (2004) Geometric morphometrics: Ten years of progress following the ‘revolution’. Italian J Zoology 71(1): 5–16.

[16] Schäfer K, Mitteröcker P, Fink B, Bookstein FL (2009) Psychomorphospace – From Biology to Perception, and Back: Towards an Integrated Quantification of Facial Form Variation. Biol Theory 4(1): 98–106.

[17] Rohlf FJ (2000) Statistical power comparisons among alternative morphometric methods. Am J Phys Anthr 111: 463–478. 10.1002/(SICI)1096-8644(200004)111:4<463::AID-AJPA3>3.0.CO;2-B 10727966

[18] Rohlf FJ (2003) Bias and error in estimates of mean shape in geometric morphometrics. J Hum Evol 44: 665–683. 12799158 10.1016/s0047-2484(03)00047-2

[19] Buchanan B, Collard M (2010) A geometric morphometrics-based assessment of blade shape differences among Paleoindian projectile point types from western North America. J Archaeol Sci 37: 350–359 doi: 10.1016/j.jas.2009.09.047

[20] Rohlf FJ (2008) NTSYSpc: Numerical Taxonomy System, ver. 2.20. Exeter Publishing, Ltd.: Setauket, NY.

[21] Zinner D, Alberts SC, Nunn CL, Altmann J (2002) Significance of primate sexual swellings. Nature. Brief Comm 420: 142–143. 10.1038/420142a 12432379

